# Development and validation of risk prediction equations to estimate future risk of blindness and lower limb amputation in patients with diabetes: cohort study

**DOI:** 10.1136/bmj.h5441

**Published:** 2015-11-11

**Authors:** Julia Hippisley-Cox, Carol Coupland

**Affiliations:** 1 Division of Primary Care, Nottingham University, Nottingham NG2 7RD, UK

## Abstract

**Study question** Is it possible to develop and externally validate risk prediction equations to estimate the 10 year risk of blindness and lower limb amputation in patients with diabetes aged 25-84 years?

**Methods** This was a prospective cohort study using routinely collected data from general practices in England contributing to the QResearch and Clinical Practice Research Datalink (CPRD) databases during the study period 1998-2014. The equations were developed using 763 QResearch practices (n=454 575 patients with diabetes) and validated in 254 different QResearch practices (n=142 419) and 357 CPRD practices (n=206 050). Cox proportional hazards models were used to derive separate risk equations for blindness and amputation in men and women that could be evaluated at 10 years. Measures of calibration and discrimination were calculated in the two validation cohorts.

**Study answer and limitations** Risk prediction equations to quantify absolute risk of blindness and amputation in men and women with diabetes have been developed and externally validated. In the QResearch derivation cohort, 4822 new cases of lower limb amputation and 8063 new cases of blindness occurred during follow-up. The risk equations were well calibrated in both validation cohorts. Discrimination was good in men in the external CPRD cohort for amputation (D statistic 1.69, Harrell’s C statistic 0.77) and blindness (D statistic 1.40, Harrell’s C statistic 0.73), with similar results in women and in the QResearch validation cohort. The algorithms are based on variables that patients are likely to know or that are routinely recorded in general practice computer systems. They can be used to identify patients at high risk for prevention or further assessment. Limitations include lack of formally adjudicated outcomes, information bias, and missing data.

**What this study adds** Patients with type 1 or type 2 diabetes are at increased risk of blindness and amputation but generally do not have accurate assessments of the magnitude of their individual risks. The new algorithms calculate the absolute risk of developing these complications over a 10 year period in patients with diabetes, taking account of their individual risk factors.

**Funding, competing interests, data sharing** JH-C is co-director of QResearch, a not for profit organisation which is a joint partnership between the University of Nottingham and Egton Medical Information Systems, and is also a paid director of ClinRisk Ltd. CC is a paid consultant statistician for ClinRisk Ltd.

## Introduction

Diabetes is associated with macrovascular complications including an increased risk of coronary heart disease or stroke and microvascular complications such as kidney failure, blindness, and amputation.^1^ ^2^ ^3^ Intensive control of risk factors such as glycated haemoglobin and systolic blood pressure lowers the incidence of microvascular disease in type 1 and type 2 diabetes.^2^ ^4^ ^5^ ^6^ Tight control of blood parameters is the cornerstone of national guidance, national audits, and quality improvement incentives schemes.^3^ ^7^ ^8^ ^9^ However, patients need good quality information on how likely they are to develop complications and the expected risks and benefits from interventions to reduce the risk, as very few patients are able to quantify this accurately.^10^ Guidelines for cardiovascular disease recommend the use of calculators such as QRISK2 to estimate the absolute risk of cardiovascular disease while taking account of patients’ characteristics.^7^ Although QRISK2 and related tools can be used to assess individualised absolute risk of cardiovascular disease, stroke, and kidney failure in patients with diabetes,^11^ ^12^ ^13^ no tools are available to calculate the risk of other complications such as amputation or blindness. This is important because these are the complications that patients with diabetes fear most and that most impair their quality of life.^14^ They are also the complications for which patients are most likely to overestimate their risk and overestimate the benefits of intensive treatment.^10^

The UK Prospective Diabetes Study (UKPDS) is a source of information on the incidence of amputation and blindness, based on a cohort that originated from a trial of 5102 patients aged 25-65 with newly diagnosed type 2 diabetes recruited between 1977 and 1991 and followed up until 1997.^5^ However, very few patients in the cohort developed blindness (n=116) or needed amputation (n=45) during follow-up.^1^ ^6^ Also, the generalisability of the cohort is limited because of its historical nature and exclusion of people aged over 65 and those with various comorbidities.

We aimed to derive and externally validate risk prediction equations to quantify absolute 10 year risks of blindness and amputation in patients with diabetes by using variables recorded in their primary care electronic record. Our intention was to provide a readily accessible method to quantify an individual patient’s absolute risks of blindness and amputation to complete a risk profile for patients with diabetes. This information could be used to provide better information for patients and doctors and to prioritise those patients at the highest levels of risk to inform treatment decisions and for closer management of modifiable risk factors.

## Methods

### Study design and data source

We did a cohort study using the UK QResearch database (version 39, www.qresearch.org) to derive and validate the risk equations in a large population of primary care patients with diabetes. We also carried out an external validation using the Clinical Practice Research Datalink (CPRD) database. QResearch is a continually updated, patient level, pseudonymised database with data extending back to 1989. It includes clinical and demographic data from more than 1000 general practices covering a population of more than 20 million patients, collected in the course of routine healthcare. The primary care data include demographic information, diagnoses, prescriptions, referrals, laboratory results, and clinical values. Diagnoses, symptoms, and clinical values are recorded using the Read code classification.^15^ QResearch has been used for a wide range of clinical research, including the development and validation of risk prediction models.^11^ ^12^ ^16^ The primary care data are linked at individual patient level to Hospital Episode Statistics (HES) and to mortality records from the Office for National Statistics (ONS). HES provides details of all National Health Service (NHS) inpatient admissions since 1997, including primary and secondary causes coded using the ICD-10 (international classification of diseases, 10th revision) classifications and operations and procedures coded using the fourth revision of the Office of Population, Censuses and Surveys Classification of Surgical Operations and Procedures (OPCS-4). ONS provides details of all deaths in England with primary and underlying causes, also coded using the ICD-10 classification. Patients’ records are linked using a project specific pseudonymised NHS number, which is valid and complete for 99.8% of primary care patients, 99.9% of ONS mortality records, and 98% of hospital admissions records.^1^

We included all QResearch practices in England that had been using their Egton Medical Information Systems (EMIS) computer system for at least a year. The EMIS computer system is the predominant commercial system used by 55% of family doctors in the United Kingdom for routine recording of health data for individual patients (www.emishealth.com/). We randomly allocated three quarters of these practices to the derivation dataset and the remaining quarter to a validation dataset. In both datasets, we identified open cohorts of patients aged 25-84 years registered with eligible practices between 1 January 1998 and 31 July 2014. We then selected patients with diabetes if they had a Read code for diabetes or more than one prescription for insulin or oral hypoglycaemics. We classified patients as having type 1 diabetes if their diabetes had been diagnosed before they were 35 years of age and was treated with insulin^17^; we classified all remaining patients as having type 2 diabetes. We excluded patients without a postcode related deprivation score. We determined an entry date to the cohort for each patient, which was the latest of the date of diagnosis of diabetes, 25th birthday, date of registration with the practice plus one year, date on which the practice computer system was installed plus one year, and the beginning of the study period (1 January 1998). Patients were censored at the earliest date of the diagnosis of the relevant complication (blindness or lower limb amputation), death, de-registration with the practice, last upload of computerised data, or the study end date (1 August 2014).

We did an external validation using general practices in England contributing to the CPRD database. This is a similar database to QResearch except that it is derived from practices using a different clinical computer system. We used the subset of 357 CPRD practices linked to ONS mortality and hospital admission data. We used the same definitions for selecting a validation cohort as for QResearch, except that the study end date was 1 August 2012, the latest date for which linked data were available.

### Outcomes

We had two outcomes of interest: lower limb amputation based on a recorded diagnosis or procedure (including above knee and below knee amputations) and blindness (including blindness in one or both eyes, registered blind, severe visual impairment). We classified patients as having the outcome if a record of the relevant diagnosis was present in their primary care record, their linked hospital record, or the ONS mortality record. We used Read codes to identify recorded diagnoses from the primary care record. We used ICD-10 clinical codes and OPCS-4 procedure codes to identify incident cases of each outcome from hospital records.^18^ We used ICD-10 codes to identify cases from either the primary or underlying cause of death as recorded on the linked ONS mortality record. The web appendix gives a list of the Read codes, OPCS-4, and ICD-10 codes used. We used the earliest recorded date of the relevant diagnosis or procedure on any of the three data sources as the index date for the diagnosis. Patients with lower limb amputation at baseline were excluded from the cohort for the analyses of lower limb amputations during follow-up and similarly for blindness.

### Predictor variables

We examined the following predictor variables based on established risk factors for vascular disease^1^ ^6^ ^11^ ^19^ ^20^ ^21^: age at cohort entry (continuous),^22^ type of diabetes (type 1 or type 2),^2^ number of years since diagnosis of diabetes (<1, 1-3, 4-6, 7-10, ≥11 years), smoking status (non-smoker; ex-smoker; light (1-9 cigarettes/day), moderate (10-19/day), heavy (≥20/day) smoker),^22^ ethnic group (white/not recorded, Indian, Pakistani, Bangladeshi, other Asian, black Caribbean, black African, Chinese, other),^19^ Townsend deprivation score (continuous),^11^ ^21^ glycated haemoglobin (HbA_1c_ mmol/mol, continuous)^1^ ^22^ ^23^ ^24^, systolic blood pressure (mm Hg, continuous),^6^ ^22^ body mass index (kg/m^2^, continuous), total serum cholesterol/high density lipoprotein cholesterol ratio (continuous),^11^ atrial fibrillation,^11^ congestive cardiac failure, cardiovascular disease, treated hypertension,^11^ peripheral vascular disease,^21^ chronic renal disease, rheumatoid arthritis,^11^ and proliferative retinopathy or maculopathy.

For each of the continuous clinical variables, we used the value recorded closest to the baseline cohort entry date out of all those recorded before the baseline date or within the six months after this date. All other predictor variables were based on the latest information recorded in the primary care record before entry to the cohort. The United Kingdom now uses the SI unit of millimoles of HbA_1c_ per mole of haemoglobin (mmol/mol) instead of the percentage.^25^ We converted historical values recorded in percentages to mmol/mol.^26^

### Derivation of models

We used established methods to develop risk prediction equations for lower limb amputation and blindness in the derivation cohort.^11^ ^12^ We derived separate equations for men and women. Initially, we used complete case analyses to derive fractional polynomial terms to model non-linear risk relations with continuous variables if appropriate (age, body mass index, systolic blood pressure, serum cholesterol/high density lipoprotein ratio, HbA_1c_).^27^ We then used multiple imputation to replace missing values for continuous values and smoking status and used these values in our main analyses.^28^ ^29^ ^30^ We included all the candidate predictor variables listed above in the multiple imputation models, along with the log of survival time and the censoring indicator. We log transformed body mass index, HbA_1c_, cholesterol, and high density lipoprotein cholesterol before imputation, as they had positively skewed distributions. We carried out 10 imputations to improve the statistical efficiency of the estimates.^31^ We used Cox’s proportional hazards models to estimate the coefficients for each risk factor for both of our outcomes by using the fractional polynomial terms obtained from the complete case analyses. We used Rubin’s rules to combine the regression coefficients across the imputed datasets.^32^ We fitted full models initially and then retained variables if they had a hazard ratio below 0.80 or above 1.20 (for binary variables) and were statistically significant at the 0.05 level*.* We examined interactions between predictor variables and age and included these if they were significant and plausible (that is, similar in direction for both men and women and consistent with the literature) and they improved model fit. We assessed model fit by measuring the Akaike information criterion and bayesian information criterion values for each imputed set of data.

We used the regression coefficients for each variable from the final model as weights, which we combined with the baseline survivor function evaluated up to 15 years to derive risk equations over a period of 15 years of follow-up.^33^ This enabled us to derive absolute risk estimates for each year of follow-up, with a specific focus on 10 year risk estimates. We estimated the baseline survivor function on the basis of zero values of centred continuous variables, with all binary predictor values set to zero.

### Validation of models

We used multiple imputation in the two validation cohorts to replace missing values for continuous variables and smoking status. We carried out 10 imputations. We applied the risk equations for men and women obtained from the derivation cohort to the validation cohorts and calculated measures of discrimination. We calculated R^2^ values (explained variation in time to diagnosis of outcome),^34^ D statistics (a measure of discrimination for which higher values indicate better discrimination),^35^ and Harrell’s C statistics (an extension of the receiver operating characteristic statistic to survival data)^36^ over 10 years and combined these model performance measures across imputed datasets by using Rubin’s rules. We assessed calibration, comparing the mean predicted risks at 10 years with the observed risk by 10th of predicted risk. The observed risks were obtained using Kaplan-Meier estimates evaluated at 10 years. We applied the risk equations to the validation cohorts to define thresholds for the 10% and 20% of patients at the highest estimated risk at 10 years and calculated sensitivity, specificity, and observed risks for these thresholds. 

We used all the available data for eligible patients on each database to maximise power and generalisability. We used Stata (version 13.1) for all analyses. We adhered to the TRIPOD statement for reporting.^37^

### Patient involvement

Patients were not involved in setting the research question, the outcome measures, or the design or implementation of the study. Patient representatives from the QResearch Advisory Board have written the information for patients on the QResearch website about the use of the database for research. They have also advised on dissemination, including the use of lay summaries describing the research and its results.

## Results

### Overall study population

Overall, 1017 QResearch practices in England met our inclusion criteria, of which 763 were randomly assigned to the derivation dataset; the remaining 254 practices were assigned to the validation cohort. We identified 455 551 patients aged 25-84 years with diabetes in the derivation cohort. We excluded 976 (0.21%) patients without a recorded Townsend deprivation score, leaving 454 575 for the derivation analysis. We identified 142 718 patients aged 25-84 years with diabetes in the QResearch validation cohort. We excluded 299 (0.21%) patients without a recorded Townsend deprivation score, leaving 142 419 for validation analysis. We identified 206 050 patients aged 25-84 years with diabetes in the CPRD validation cohort from the 357 practices with linked Townsend scores and hospital admissions and mortality data.

### Baseline characteristics

Table 1 shows baseline characteristics of 454 575 patients with diabetes in the derivation cohort at study entry. Of these, 94% had type 2 diabetes. Just over half had been diagnosed as having diabetes less than a year before cohort entry, 17% had been diagnosed for 1-3 years, 9% for 4-6 years, 8% for 7-10 years, and 12% for 11 or more years. Smoking status was recorded in 95% of patients, ethnicity in 75%, body mass index in 90%, systolic blood pressure in 97%, HbA_1c_ in 71%, and cholesterol/high density lipoprotein cholesterol ratio in 53%. Of the 454 575 patients in the derivation cohort, 266 142 (58.6%) had missing data for at least one of these variables (including ethnicity).

**Table 1 tbl1:** Baseline characteristics of patients with diabetes aged 25-84 years in QResearch derivation cohort and both validation cohorts. Values are numbers (percentages) unless stated otherwise.

Characteristics	QResearch derivation cohort			QResearch validation cohort			CPRD validation cohort	
	Women (n=199 679)	Men (n=254 896)		Women (n=62 407)	Men (n=80 012)		Women (n=90 280)	Men (n=115 770)
Type 2 diabetes	188 086 (94.2)	241 058 (94.6)		58 852 (94.3)	75 717 (94.6)		85 361 (94.6)	109 540 (94.6)
Type 1 diabetes	11 593 (5.8)	13 838 (5.4)		3555 (5.7)	4295 (5.4)		4919 (5.4)	6230 (5.4)
Years since diagnosis:	****	****	****	****	****	****	****	****
Newly diagnosed (<1 year)	108 040 (54.1)	137 725 (54.0)		34 900 (55.9)	44 412 (55.5)		48 913 (54.2)	62 922 (54.4)
1-3 years	33 256 (16.7)	43 790 (17.2)		9819 (15.7)	12 902 (16.1)		14 912 (16.5)	19 345 (16.7)
4-6 years	18 826 (9.4)	23 855 (9.4)		5552 (8.9)	7159 (8.9)		8283 (9.2)	10 535 (9.1)
7-10 years	15 895 (8.0)	19 950 (7.8)		4824 (7.7)	6256 (7.8)		7285 (8.1)	9255 (8.0)
>10 years	23 662 (11.9)	29 576 (11.6)		7312 (11.7)	9283 (11.6)		10 887 (12.1)	13 713 (11.8)
Mean (SD) age, years	61.5 (14.1)	59.5 (13.4)		62 (14.0)	59.9 (13.3)		62.7 (13.7)	60.4 (12.9)
Mean (SD) Townsend score	0.8 (3.4)	0.5 (3.4)		0.4 (3.3)	0.1 (3.2)		0 (3.3)	−0.4 (3.2)
Ethnicity recorded:	150 526 (75.4)	191 204 (75.0)		46 575 (74.6)	59 394 (74.2)		40 151 (44.5)	51 522 (44.5)
White/not recorded	164 366 (82.3)	214 557 (84.2)		53 760 (86.1)	70 000 (87.5)		83 962 (93.0)	108 518 (93.7)
Indian	6836 (3.4)	9027 (3.5)		1928 (3.1)	2606 (3.3)		1503 (1.7)	2036 (1.8)
Pakistani	5011 (2.5)	5744 (2.3)		854 (1.4)	1071 (1.3)		778 (0.9)	801 (0.7)
Bangladeshi	5979 (3.0)	6731 (2.6)		956 (1.5)	1028 (1.3)		268 (0.3)	321 (0.3)
Other Asian	3134 (1.6)	4017 (1.6)		1005 (1.6)	1393 (1.7)		865 (1.0)	1083 (0.9)
Caribbean	5614 (2.8)	4653 (1.8)		1578 (2.5)	1291 (1.6)		919 (1.0)	768 (0.7)
Black African	3831 (1.9)	4654 (1.8)		1004 (1.6)	1102 (1.4)		838 (0.9)	891 (0.8)
Chinese	693 (0.3)	719 (0.3)		193 (0.3)	222 (0.3)		138 (0.2)	168 (0.1)
Other	4215 (2.1)	4794 (1.9)		1129 (1.8)	1299 (1.6)		1009 (1.1)	1184 (1.0)
Smoking status recorded:	189 827 (95.1)	243 379 (95.5)		59 409 (95.2)	76 617 (95.8)		89 107 (98.7)	114 577 (99.0)
Non-smoker	118 807 (59.5)	108 368 (42.5)		36 291 (58.2)	33 839 (42.3)		43 414 (48.1)	40 977 (35.4)
Ex-smoker	41 073 (20.6)	83 683 (32.8)		13 572 (21.7)	27 231 (34.0)		14 002 (15.5)	28 100 (24.3)
Light smoker	16 090 (8.1)	30 116 (11.8)		5112 (8.2)	9080 (11.3)		4879 (5.4)	7799 (6.7)
Moderate smoker	7720 (3.9)	10 684 (4.2)		2512 (4.0)	3196 (4.0)		9772 (10.8)	12 756 (11.0)
Heavy smoker	6137 (3.1)	10 528 (4.1)		1922 (3.1)	3271 (4.1)		5931 (6.6)	11 363 (9.8)
Amount smoked not recorded	NA	NA		NA	NA		11 109 (12.3)	13 582 (11.7)
Medical conditions at baseline:	****	****	****	****	****	****	****	****
Atrial fibrillation	7995 (4.0)	11009 (4.3)		2684 (4.3)	3626 (4.5)		3952 (4.4)	5273 (4.6)
Congestive cardiac failure	6783 (3.4)	9986 (3.9)		2255 (3.6)	3136 (3.9)		3504 (3.9)	4641 (4.0)
Cardiovascular disease	31 729 (15.9)	55 262 (21.7)		10 170 (16.3)	17 453 (21.8)		16 188 (17.9)	26 826 (23.2)
Treated hypertension	78 323 (39.2)	85 634 (33.6)		24 451 (39.2)	26 721 (33.4)		31 477 (34.9)	32 465 (28.0)
Peripheral vascular disease	5242 (2.6)	10 380 (4.1)		1692 (2.7)	3257 (4.1)		2846 (3.2)	5344 (4.6)
Chronic renal disease	2325 (1.2)	2857 (1.1)		718 (1.2)	905 (1.1)		930 (1.0)	1185 (1.0)
Rheumatoid arthritis	7458 (3.7)	4651 (1.8)		2204 (3.5)	1477 (1.8)		1976 (2.2)	1206 (1.0)
Proliferative retinopathy or maculopathy	5531 (2.8)	7657 (3.0)		1653 (2.6)	2162 (2.7)		1319 (1.5)	1913 (1.7)
Existing blindness	3416 (1.7)	3701 (1.5)		1126 (1.8)	1169 (1.5)		1789 (2.0)	1656 (1.4)
Existing lower limb amputation	1010 (0.5)	2073 (0.8)		346 (0.6)	728 (0.9)		455 (0.5)	1013 (0.9)
Clinical values at baseline:	****	****	****	****	****	****	****	****
HbA_1c_ recorded	141 005 (70.6)	180 594 (70.9)		43 575 (69.8)	56 107 (70.1)		51 725 (57.3)	67 013 (57.9)
Mean (SD) HbA_1c_, mmol/mol	61.4 (20.8)	63 (22.0)		61.1 (20.8)	62.9 (21.9)		60.8 (21.1)	62.6 (22.0)
Body mass index recorded	179 818 (90.1)	232 298 (91.1)		55 892 (89.6)	72 979 (91.2)		82 814 (91.7)	107 778 (93.1)
Mean (SD) body mass index	31.1 (6.3)	29.8 (5.3)		31.2 (6.4)	29.9 (5.3)		30.9 (6.3)	29.7 (5.3)
Cholesterol ratio recorded	105 436 (52.8)	138 385 (54.3)		33 392 (53.5)	43 988 (55.0)		35 174 (39.0)	46 530 (40.2)
Mean (SD) cholesterol/high density lipoprotein cholesterol ratio	4.1 (1.4)	4.5 (1.5)		4.1 (1.4)	4.5 (1.5)		4.2 (1.5)	4.5 (1.6)
Systolic blood pressure recorded	194 001 (97.2)	246 991 (96.9)		60 728 (97.3)	77 707 (97.1)		88 792 (98.4)	113 582 (98.1)
Mean (SD) systolic blood pressure, mm Hg	139.3 (20.0)	138.4 (18.6)		139.8 (20.0)	138.6 (18.6)		141.4 (20.6)	140 (19.0)

NA=not applicable.

Baseline characteristics for patients in the QResearch validation cohort were similar to corresponding values in the derivation cohort (table 1). Of the 142 419 patients in the QResearch validation cohort, 83 403 (58.6%) had missing data for at least one variable. Baseline characteristics of the CPRD validation cohort were also similar, except that the recording of ethnicity (45%), cholesterol/high density lipoprotein cholesterol ratio (40%), and HbA_1c_ (58%) was substantially lower in CPRD than in QResearch. Of the 206 050 patients in the CPRD validation cohort, 166 648 (80.9%) had missing data for at least one variable.

### Primary outcomes of amputation and blindness

Table 2 shows the number of incident cases of each outcome during follow-up and the age standardised incidence rates in each cohort. In the QResearch derivation cohort, 4822 cases of amputation and 8063 cases of blindness occurred. In addition, 1524 cases of amputation and 2651 cases of blindness occurred in the QResearch validation cohort, and 2294 cases of amputation and 2845 cases of blindness occurred in the CPRD validation cohort. The rate of blindness was lower in men in CPRD (2.33 per 1000 person years) than in both QResearch cohorts (3.03 per 1000 person years) and was also lower in women in CPRD, but rates of amputation were similar.

**Table 2 tbl2:** Numbers of incident cases* of blindness and lower limb amputation during follow-up and age standardised incidence rates per 1000 person years in men and women with diabetes aged 25-84 years in derivation cohort and validation cohorts

	QResearch derivation cohort			QResearch validation cohort			CPRD validation cohort	
	Cases	Rate† per 1000 person years (95% CI)		Cases	Rate† per 1000 person years (95% CI)		Cases	Rate† per 1000 person years (95% CI)
**Women**								
Amputation	1541	1.34 (1.27 to 1.41 )		482	1.32 (1.20 to 1.44 )		675	1.32 (1.22 to 1.42 )
Blindness	4074	3.43 (3.33 to 3.54 )		1365	3.59 (3.40 to 3.79 )		1487	2.78 (2.64 to 2.93 )
**Men**								
Amputation	3281	2.36 (2.28 to 2.44 )		1042	2.33 (2.19 to 2.47 )		1619	2.66 (2.53 to 2.79 )
Blindness	3989	3.03 (2.93 to 3.12 )		1286	3.04 (2.88 to 3.21 )		1358	2.33 (2.20 to 2.45 )

*Patients with existing diagnoses of each complication at baseline were dropped from relevant cohort.

†Rates were directly age standardised to overall age distribution of patients aged 25-84 within QResearch derivation cohort in 5 year age bands.

### Predictor variables

Table 3 shows the adjusted hazard ratios for variables in the final models for men and women in the derivation cohort.

**Table 3 tbl3:** Adjusted hazard ratios for blindness and lower limb amputation in men and women in derivation cohort

Characteristic	Adjusted hazard ratio (95%CI)	
	Women	Men
Amputation*		
Townsend deprivation score†	1.10 (1.01 to 1.19 )	1.29 (1.22 to 1.36 )
Duration of diabetes:	****	****
Newly diagnosed (<1 year)	1	1
1-3 years	1.59 (1.36 to 1.85 )	1.68 (1.51 to 1.87 )
4-6 years	1.69 (1.42 to 2.01 )	2.03 (1.81 to 2.28 )
7-10 years	2.37 (2.01 to 2.79 )	2.67 (2.39 to 3.00 )
>10 years	3.30 (2.89 to 3.78 )	3.49 (3.15 to 3.86 )
Smoking status:	****	****
Non-smoker	1	1
Ex-smoker	1.08 (0.94 to 1.24 )	0.94 (0.87 to 1.03 )
Light smoker	1.59 (1.34 to 1.88 )	1.28 (1.14 to 1.43 )
Moderate smoker	1.58 (1.25 to 1.99 )	1.15 (0.96 to 1.37 )
Heavy smoker	1.89 (1.49 to 2.41 )	1.26 (1.06 to 1.49 )
Ethnicity:	****	****
White/not recorded	1	1
Indian	0.44 (0.28 to 0.68 )	0.42 (0.32 to 0.55 )
Pakistani	0.72 (0.47 to 1.12 )	0.40 (0.28 to 0.58 )
Bangladeshi	0.29 (0.15 to 0.56 )	0.12 (0.07 to 0.22 )
Other Asian	0.70 (0.39 to 1.27 )	0.42 (0.26 to 0.67 )
Caribbean	0.87 (0.65 to 1.18 )	0.49 (0.36 to 0.66 )
Black African	0.92 (0.55 to 1.54 )	0.38 (0.23 to 0.61 )
Chinese	0.50 (0.12 to 1.99 )	0.35 (0.11 to 1.09 )
Other	0.70 (0.44 to 1.10 )	0.63 (0.45 to 0.87 )
Comorbidity:	****	****
Type 1 diabetes (versus type 2)	NS	1.26 (1.09 to 1.45 )
Rheumatoid arthritis	1.50 (1.19 to 1.90 )	1.39 (1.11 to 1.75 )
Atrial fibrillation	NS	1.26 (1.07 to 1.49 )
Congestive cardiac failure	1.79 (1.44 to 2.22 )	1.34 (1.14 to 1.58 )
Peripheral vascular disease	4.26 (3.63 to 4.99 )	3.16 (2.84 to 3.51 )
Chronic renal disease	2.68 (1.96 to 3.66 )	2.26 (1.80 to 2.85 )
**Blindness‡**		
Cholesterol/HDL ratio§	1.06 (1.03 to 1.09 )	1.03 (1.00 to 1.06 )
Townsend deprivation score†	1.21 (1.15 to 1.27 )	1.33 (1.27 to 1.39 )
Duration of diabetes:	****	****
Newly diagnosed (<1 year)	1	1
1-3 years	1.36 (1.25 to 1.49 )	1.40 (1.28 to 1.54 )
4-6 years	1.51 (1.36 to 1.67 )	1.42 (1.28 to 1.58 )
7-10 years	1.72 (1.55 to 1.91 )	1.57 (1.41 to 1.76 )
>10 years	2.17 (1.97 to 2.38 )	2.09 (1.90 to 2.29 )
Comorbidity:	****	****
Type 1 diabetes (versus type 2)	1.50 (1.26 to 1.78 )	1.44 (1.22 to 1.70 )
Chronic renal disease	1.49 (1.17 to 1.89 )	2.57 (1.88 to 3.52 )
Proliferative retinopathy/maculopathy	2.67 (2.37 to 3.02 )	2.93 (2.61 to 3.29 )

HDL=high density lipoprotein cholesterol; NS=not significant.

*Amputation model in women also included terms for age (linear), systolic blood pressure (2 fractional polynomial (FP) terms: −1, −0.5), HbA_1c_ (2 FP terms: 3, 3); amputation model in men included terms for age (linear), systolic blood pressure (2 FP terms: −2, 0.5), HbA_1c_ (2 FP terms: 2, 2).

†Townsend deprivation score ranges between −7 (most affluent) and 11 (most deprived); adjusted hazard ratio is per 5 unit increase.

‡Blindness model in women also included terms for age (2 FP terms: 2, 2), systolic blood pressure (linear), HbA_1c_ (2 FP terms: 2, 2); model in men also included terms for age (2 FP terms: 2, 2), systolic blood pressure (2 FP terms: 1, 2), HbA_1c_ (2 FP terms: −2, −2); there was an interaction between age and renal disease in men.

§Adjusted hazard ratio is per unit increase.

#### Lower limb amputation

The final model for lower limb amputation in women included age, systolic blood pressure, HbA_1c_, deprivation, duration of diabetes, smoking status, ethnicity, rheumatoid arthritis, congestive cardiac failure, peripheral vascular disease, and chronic renal disease. The final model in men also included type of diabetes and atrial fibrillation. Body mass index and the serum cholesterol/high density lipoprotein cholesterol ratio were not significantly associated with risk in men or women. Increasing duration of diabetes was associated with an increased risk of lower limb amputation in men and women. Increasing levels of smoking were associated with an increased risk of amputation; the association was more marked in women than in men. For heavy smokers compared with non-smokers, a 1.9-fold increase in risk of amputation was seen for women and a 1.3-fold increased risk for men. South Asian ethnic groups had a lower risk compared with people whose ethnic group was either white or not recorded; Caribbean and black African men also had lower risks. Pre-existing peripheral vascular disease was associated with the highest risks (fourfold in women and threefold in men), followed by chronic renal disease (2.7-fold in women and 2.3-fold in men).

Figures 1, 2, and 3 show adjusted hazard ratios for age, HbA_1c_, and systolic blood pressure. Increasing values of age, HbA_1c_, and systolic blood pressure, were associated with an increased risk of lower limb amputation in men and women.

**Figure fig1:**
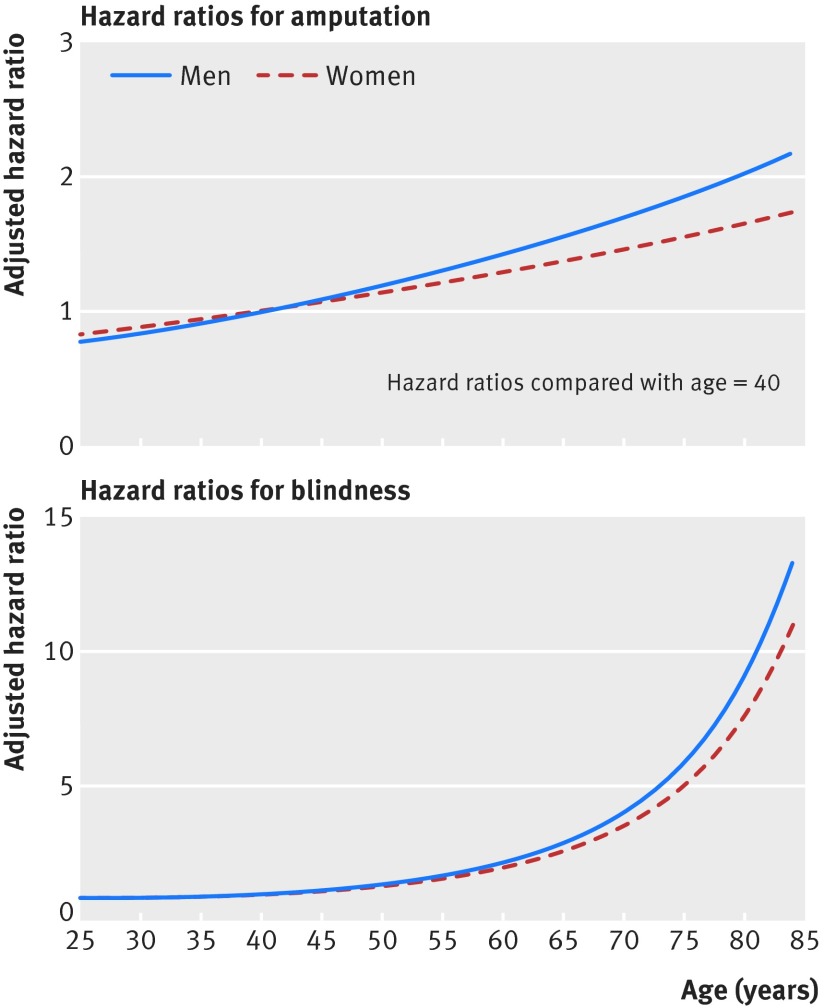
**Fig 1** Adjusted hazard ratios for blindness and lower limb amputation by age in derivation cohort

**Figure fig2:**
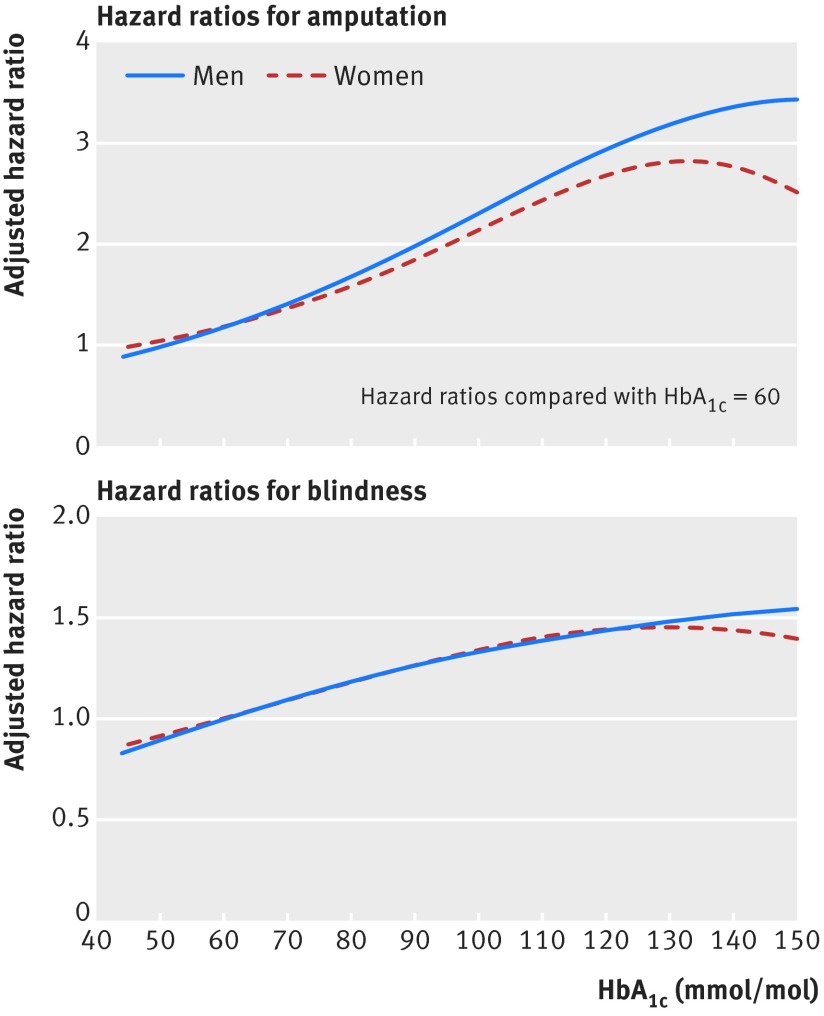
**Fig 2** Adjusted hazard ratios for blindness and lower limb amputation by HbA_1c_ in derivation cohort

**Figure fig3:**
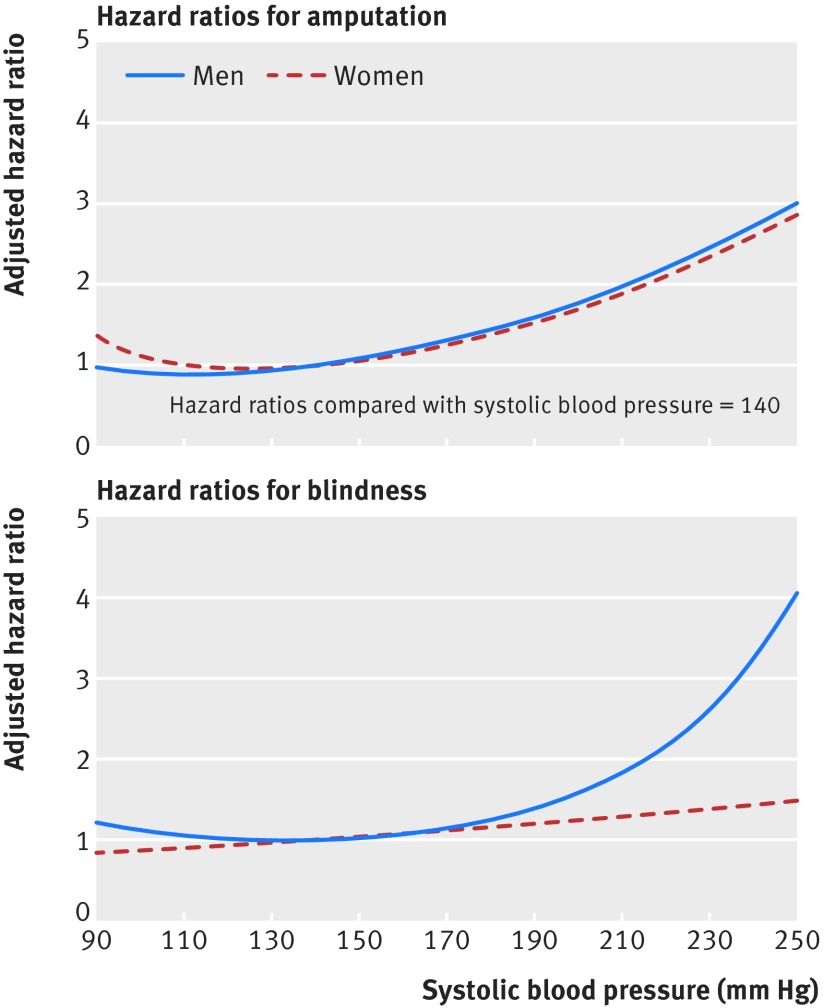
**Fig 3** Adjusted hazard ratios for blindness and lower limb amputation by systolic blood pressure in derivation cohort

#### Blindness

The final models for blindness in men and women included age, cholesterol/high density lipoprotein cholesterol ratio, systolic blood pressure, HbA_1c_, deprivation, duration of diabetes, type of diabetes, chronic renal disease, and existing proliferative retinopathy or maculopathy. Body mass index and smoking status were not significantly associated with risk. Increasing values of age, HbA_1c_, and systolic blood pressure were associated with an increased risk of blindness (figures 1, 2, and 3). Increasing values of the serum cholesterol/high density lipoprotein cholesterol ratio were also associated with an increased risk of blindness. Increasing duration of diabetes was associated with increased risk despite adjustment for age and other risk factors. We found a significant interaction between renal disease and age. Pre-existing proliferative retinopathy or maculopathy was the strongest risk factor, with a 2.7-fold increase for women and a 2.9-fold increase for men.

#### Web calculator

The web calculator that implements the risk equations for the final models can be found at qdiabetes.org/amputation-blindness/index.php, along with the open source software which includes the equations (published separately, as these will be updated over time as newer data becomes available).

### Validation

#### Discrimination

Table 4 shows the performance of each equation in both validation cohorts. For men in the CPRD cohort, the equations explained 40.6% of the variation in time to diagnosis of amputation and 31.9% for blindness, and discrimination was good for amputation (D statistic 1.69, Harrell’s C statistic 0.77) and blindness (D statistic 1.40, Harrell’s C statistic 0.73). The results for women in the CPRD cohort were very similar to those for men. The results for both sexes in the CPRD cohort were similar to those for the QResearch validation cohort, although the point estimates for CPRD tended to be marginally higher.

**Table 4 tbl4:** Performance of equations in men and women in CPRD validation cohort and QResearch validation cohort

Statistic	Mean (95% CI)	
	CPRD validation cohort	QResearch validation cohort
**Women**		
Amputation:	****	****
D statistic*	1.61 (1.45 to 1.77 )	1.30 (1.14 to 1.47 )
R^2 ^(%)†	38.22 (33.61 to 42.83 )	28.90 (23.70 to 34.10 )
Harrell’s C statistic‡	0.762 (0.735 to 0.789)	0.700 (0.670 to 0.731)
Blindness:		
D statistic*	1.36 (1.27 to 1.46 )	1.32 (1.23 to 1.42 )
R^2 ^(%)†	30.78 (27.94 to 33.63 )	29.44 (26.50 to 32.39 )
Harrell’s C statistic‡	0.733 (0.719 to 0.747)	0.725 (0.709 to 0.741)
**Men**		
Amputation:	****	****
D statistic*	1.69 (1.59 to 1.79 )	1.48 (1.38 to 1.59 )
R^2 ^(%)†	40.57 (37.70 to 43.44 )	34.42 (31.14 to 37.70 )
Harrell’s C statistic‡	0.770 (0.755 to 0.784)	0.748 (0.730 to 0.767)
Blindness:		
D statistic*	1.40 (1.31 to 1.49 )	1.33 (1.23 to 1.42 )
R^2 ^(%)†	31.93 (29.04 to 34.82 )	29.57 (26.53 to 32.62 )
Harrell’s C statistic‡	0.732 (0.716 to 0.747)	0.714 (0.696 to 0.731)

*A measure of discrimination that is specific to censored survival data for which higher values indicate better discrimination.

†Measures explained variation in time to diagnosis of outcome; higher values indicate that more variation is explained.

‡A measure of discrimination for which higher values indicate better discrimination.

#### Calibration

Figure 4 shows the mean predicted and observed risks of both outcomes at 10 years by 10th of predicted risk, applying the equations to men and women in the QResearch validation cohort. Figure 5 shows comparable results for the CPRD cohort. We found close correspondence between the mean predicted risks and the observed risks within each model 10th, indicating that the equations were well calibrated across both validation cohorts.

**Figure fig4:**
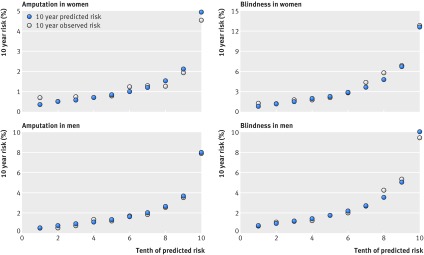
**Fig 4** Mean predicted risks and observed risks of blindness and lower limb amputation at 10 years by 10th of predicted risk, applying equations to all men and women in QResearch validation cohort

**Figure fig5:**
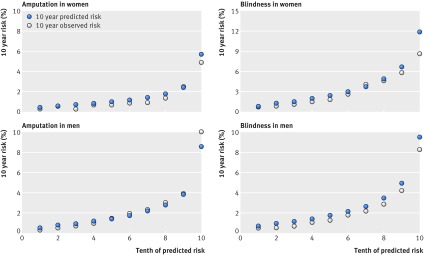
**Fig 5** Mean predicted risks and observed risks of blindness and lower limb amputation at 10 years by 10th of predicted risk, applying equations to all men and women in CPRD validation cohort

#### Performance at threshold for 10% and 20% of patients at highest risk

Table 5 shows the sensitivity, specificity, and observed risk for the 10% and 20% of men and women at the highest predicted risk of each outcome for both validation cohorts for illustrative purposes. For example, when we used a 10 year risk threshold of 3.2% for amputation in men in CPRD to identify the 20% at highest predicted risk, the sensitivity was 58%, the specificity was 80.5%, and the observed risk was 7%.

**Table 5 tbl5:** Performance of each model in both QResearch and CPRD validation cohorts based on 10% and 20% of patients at highest predicted risk

	QResearch cohort					CPRD cohort			
	Cut-off (%) for 10 year risk*	Sensitivity (%)	Specificity (%)	Observed risk (%)		Cut-off (%) for 10 year risk*	Sensitivity (%)	Specificity (%)	Observed risk (%)
**Women**									
Amputation (top 10%)	2.6	33.2	90.2	4.6		2.9	39.4	90.2	4.9
Amputation (top 20%)	1.8	48.1	80.2	3.2		2.0	59.8	80.3	3.7
Blindness (top 10%)	8.1	27.9	90.4	12.8		8.0	25.7	90.2	8.7
Blindness (top 20%)	5.6	45.1	80.5	9.6		5.6	44.3	80.4	7.2
**Men**									
Amputation (top 10%)	4.5	37.5	90.3	7.9		4.8	41.9	90.4	10.2
Amputation (top 20%)	3.0	53.5	80.4	5.7		3.2	58.0	80.5	7.0
Blindness (top 10%)	6.2	27.6	90.2	9.5		6.0	31.5	90.2	8.3
Blindness (top 20%)	4.1	45.9	80.4	7.2		4.1	49.1	80.3	6.1

*Risk threshold for 10% or 20% of patients at highest predicted risk of outcome over 10 years.

### Implementation

Figure 6 shows a clinical example of the implementation of the equations using the web calculator (qdiabetes.org/amputation-blindness/index.php). The example is for a 50 year old female non-smoker with newly diagnosed type 2 diabetes and an HbA_1c_ of 65 mmol/mol, a cholesterol/high density lipoprotein cholesterol ratio of 2, and a systolic blood pressure of 140 mm Hg. Her 10 year risk of blindness is 1%, and her risk of amputation is 0.5%.

**Figure fig6:**
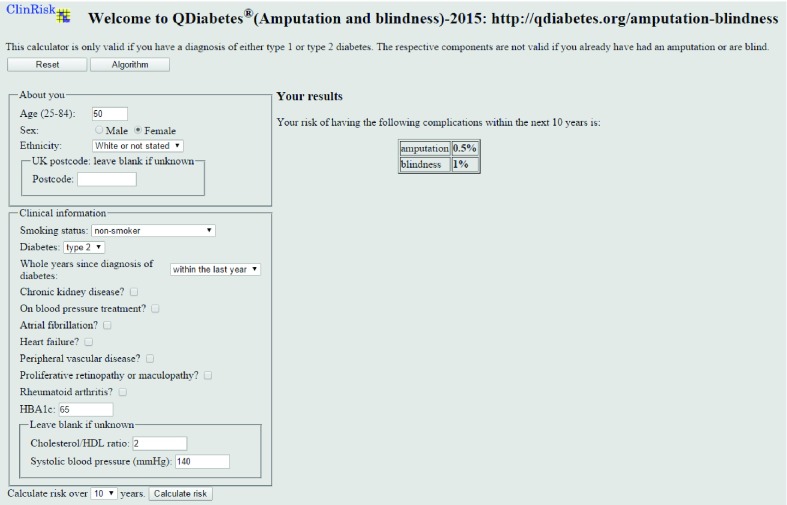
**Fig 6** Web calculator applied to example female patient

Figure 7 shows the results for a 75 year old man, diagnosed as having type 2 diabetes 10 years ago, who is a moderate smoker and has chronic kidney disease, an HbA_1c_ of 70 mmol/mol, a cholesterol/high density lipoprotein cholesterol ratio of 4, and a systolic blood pressure of 160 mm Hg. His 10 year risk of blindness is 14.7%, and his risk of amputation is 12.1%.

**Figure fig7:**
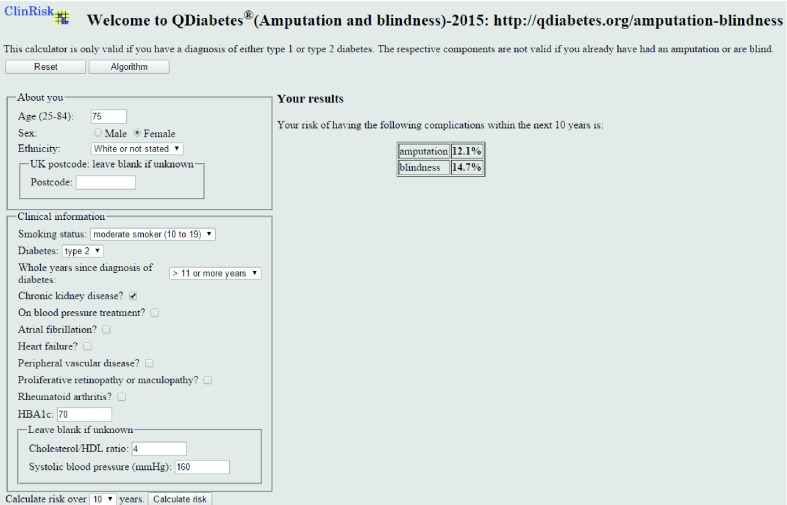
**Fig 7** Web calculator applied to example male patient

## Discussion

We have developed and externally validated risk prediction equations to quantify the absolute risks of blindness and lower limb amputation over 10 years in men and women with type 1 and type 2 diabetes. The equations are well calibrated and have good discrimination, with C statistic values of at least 0.73 in the external CPRD validation cohort. To our knowledge, these are the first tools for predicting the 10 year risk of both blindness and amputation, two of the complications that most concern patients with diabetes and affect quality of life.

### Clinical implications

These algorithms are designed to provide better information for patients and doctors on the absolute risks of blindness and amputation, to inform management decisions. Patients with diabetes tend to overestimate their risk of complications and also overestimate the benefits of treatment.^10^ For example, in one study, patients believed that they were 1.5 times more likely to become blind and 13 times more likely to have a lower leg amputation than estimates of absolute risk based on the DCCT trial.^2^ ^10^ Some people may argue that overestimating the risk of complications might result in patients being more likely to take intensive treatment. However, from a holistic and ethical point of view, more accurate individualised information on the risk of complications may help patients to make more informed decisions about the balance of risks and benefits of treatment options reflecting their own values and choices. Overestimation of the risk of complications might lead to increased levels of anxiety and depression, which could negatively affect quality of life. This is especially important as patients with diabetes are more likely than the general population to experience anxiety and depression.^38^

For clinicians and the health service, more accurate methods for stratifying patients according to their absolute risk of complications could enable screening programmes to be tailored to an individual’s level of risk and support the more rational use of scarce resources. For example, blindness can be prevented by screening for and treatment of retinopathy,^39^ and patients at high risk of blindness might need retinal screening more often than once a year. Those at higher risk of amputation might benefit from a proactive targeted programme to prevent lower extremity amputation (including more frequent checks, tailored patient education, specially designed protective footwear, and early reporting of foot injuries), as this has been shown to substantially reduce the risk of emergency admissions, use of antibiotics, foot operations, and lower limb amputation compared with usual practice.^40^ ^41^ Better information on the absolute risk of individual complications could also prompt more intensive treatment of modifiable risk factors—such as lowering of HbA_1c_ and tighter blood pressure control—which are generally considered to reduce the risk of microvascular complications such as blindness.^2^ ^5^ ^42^

### Comparisons with literature

The incidence rates of amputation and blindness are comparable to the amputation rate of 1.6 per 1000 patient years and blindness rate of 3.5 per 1000 patient years reported by the UKPDS.^5^ However, our study is approximately 100-fold larger than the UKPDS, with almost 5000 incident amputations and more than 8000 cases of recorded blindness in the derivation cohort, and it is 10 times larger than the US hospital based cohort study reported by Zhao et al.^24^ Our study is also more recent than the UKPDS, which started almost 40 years ago and ended almost 20 years ago.^5^ Our study included patients with prevalent type 1 and type 2 diabetes as well as those with a new diagnosis, enabling us to account for the important contribution of duration of diabetes to risk and to ensure that the results can be applied to patients with either newly diagnosed or prevalent diabetes.

We included established risk factors in our equations and report hazard ratios similar in both magnitude and direction to those reported elsewhere for lower limb amputation,^1^ progression of retinopathy, and blindness,^1^ ^20^ which increases the clinical face validity of the equations. As in the UKPDS,^6^ increased systolic blood pressure was associated with increased risks of blindness and lower limb amputation,^20^ and increased levels of HbA_1c_ were associated with increased risks of blindness and amputation when compared over equivalent ranges.^1^ ^24^ Deprivation and smoking were associated with an increased risk of amputation in our study and others.^21^ However, smoking was not associated with an increased risk of blindness in our study, which is consistent with other research.^20^ Non-white ethnic groups had lower risks of lower limb amputation compared with the white group. This contrasts with a US study in which black Africans had a higher risk of amputation.^19^

Three economic models have been based on the DCCT and UKPDS studies.^2^ ^5^ The CORE diabetes and Sheffield diabetes models are based on equations derived from the DCCT trial and the UKPDS study.^43^ ^44^ ^45^ The EAGLE model is based on equations derived from UKPDS and the DCCT, as well as the Wisconsin Epidemiological Study of Diabetic Retinopathy.^46^ The CORE model predicts risk of amputation,^46^ and the CORE, EAGLE, and Sheffield models predict retinopathy rather than blindness.

### Methodological considerations

The methods used to derive and validate these models are very similar to those for other risk prediction tools derived from the QResearch database, the strengths and limitations of which have been discussed in detail.^11^ ^12^ In summary, key strengths include cohort size, duration of follow-up, representativeness, and lack of selection, recall, and respondent bias. UK general practices have good levels of accuracy and completeness in recording clinical diagnoses and prescribed drugs.^47^ The QResearch database has linked hospital and mortality records for almost all patients and is therefore likely to have picked up most cases of lower limb amputation, thereby minimising ascertainment bias. The QResearch database is updated regularly, allowing us to update the algorithms over time to reflect changes in data quality, population characteristics, or requirements, thereby keeping the tools up to date.

We undertook two validations, one using a separate set of practices and patients contributing to QResearch and the other using a fully external set of practices contributing to CPRD. The results of both validations were extremely similar, which is consistent with previous validation studies showing comparable performance using different practice populations.^48^ ^49^ Although we have derived and validated the equations using UK datasets, the equations could be used internationally by using alternative deprivation scores relevant to the setting (which would need to be scaled to conform with the Townsend score). Local validation should be done to ensure good calibration and discrimination in the applicable population, as patients from different countries may have different rates of complications or distributions of risk factors.

Limitations of our study include the lack of formal adjudication of diagnoses and the potential for bias due to missing data, which we have addressed using multiple imputation. Although we have provided analysis of several thresholds for illustrative purposes, we have not provided definite comment on what threshold of absolute risk should be used to define a “high risk” group, as that would require consideration of the balance of risks and benefits for individuals and cost effectiveness analyses, which are outside the scope of this study.

### Conclusion

We have developed and validated new risk prediction equations to quantify the absolute risks of blindness and lower limb amputation in patients with diabetes. They can be used to identify patients with diabetes at high risk of these complications for further assessment. Further research is needed to evaluate the clinical outcomes and cost effectiveness of using these risk equations in primary care.

**What is known on this topic**

Patients with type 1 or type 2 diabetes are at increased risk of blindness and amputation but generally do not have an accurate assessment of the magnitude of their individual risk

**What this paper adds**

New risk prediction algorithms for blindness and amputation have been developed and externally validated
These calculate the absolute risk of developing these complications over a 10 year period in patients with diabetes, taking account of their individual risk factors
The web calculator to calculate the absolute risk of complications among patients with diabetes is available at qdiabetes.org/amputation-blindness/index.php

## Notes

We acknowledge the contribution of EMIS practices who contribute to the QResearch and EMIS for expertise in establishing, developing, and supporting the database.

Ethical approval: The project was reviewed in accordance with the QResearch agreement with NRES Committee East Midlands - Derby (reference 03/4/021). The project was reviewed by the independent scientific committee of the Clinical Research Practice Datalink (reference 13_079).

Data sharing: The equations presented in this paper will be released as open source software under the GNU lesser GPL v3. Open source software allows use without charge under the terms of the GNU lesser public license version 3. Closed source software can be licensed at a fee.

Transparency declaration: The lead author (the manuscript’s guarantor) affirms that this manuscript is an honest, accurate, and transparent account of the study being reported; that no important aspects of the study have been omitted; and that any discrepancies from the study as planned (and, if relevant, registered) have been explained.

## Notes

Cite this as: *BMJ* 2015;351:h5441
