# Supplementary material for: Development and validation of risk prediction equations to estimate future risk of blindness and lower limb amputation in patients with diabetes: cohort study
Source: BMJ. 2015 Nov 11;351:h5441. doi: 10.1136/bmj.h5441 (PMC4641884; doi:10.1136/bmj.h5441)
Supplement: Supplementary file 1 [file coxj027121.ww1_default.pdf]

Group Name	clincial cod	clinical term
Blindness (Read code)	2B6A-1	O/E - blind R-eye
Blindness (Read code)	2B6B	O/E - R-eye completely blind
Blindness (Read code)	2B6S	O/E - pinhole R-eye completely blind
Blindness (Read code)	2B7A-1	O/E - blind L-eye
Blindness (Read code)	2B7B	O/E - L-eye completely blind
Blindness (Read code)	2B7S	O/E - pinhole L-eye completely blind
Blindness (Read code)	6688	Registered partially sighted
Blindness (Read code)	6688-1	Registered partially blind
Blindness (Read code)	6689	Registered blind
Blindness (Read code)	6689-1	Registered severely sight impaired
Blindness (Read code)	668C	Certificate of vision impairment
Blindness (Read code)	8F6-1	Blind rehabilitation
Blindness (Read code)	8F61	Blind rehabilitation
Blindness (Read code)	8F62	Blind lead dog rehabilitation
Blindness (Read code)	9m08	Excluded from diabetic retinopathy screening as blind
Blindness (Read code)	EGTONBL2	Blind (subjectively)
Blindness (Read code)	EGTONVI8	Vision - blind despite any aid
Blindness (Read code)	F49	Blindness and low vision
Blindness (Read code)	F49-1	Impaired vision
Blindness (Read code)	F49-2	Low vision
Blindness (Read code)	F49-3	Partial sight
Blindness (Read code)	F49-4	Sight impaired
Blindness (Read code)	F490	Blindness, both eyes
Blindness (Read code)	F490-98	Blind/low vision - both eyes
Blindness (Read code)	F490-99	Blind - both eyes
Blindness (Read code)	F4900	Unspecified blindness both eyes
Blindness (Read code)	F4902	Better eye: near total VI, Lesser eye: unspecified
Blindness (Read code)	F4903	Better eye: near total VI, Lesser eye: total VI
Blindness (Read code)	F4904	Better eye: near total VI, Lesser eye: near total VI
Blindness (Read code)	F4905	Better eye: profound VI, Lesser eye: unspecified
Blindness (Read code)	F4906	Better eye: profound VI, Lesser eye: total VI
Blindness (Read code)	F4907	Better eye: profound VI, Lesser eye: near total VI
Blindness (Read code)	F4908	Better eye: profound VI, Lesser eye: profound VI
Blindness (Read code)	F4909	Acquired blindness, both eyes
Blindness (Read code)	F490z	Blindness both eyes NOS
Blindness (Read code)	F491	Better eye: low vision, Lesser eye: profound VI
Blindness (Read code)	F4910	One eye blind, one eye low vision
Blindness (Read code)	F4911	Better eye: severe VI, Lesser eye: blind, unspecified
Blindness (Read code)	F4912	Better eye: severe VI, Lesser eye: total VI
Blindness (Read code)	F4913	Better eye: severe VI, Lesser eye: near total VI
Blindness (Read code)	F4914	Better eye: severe VI, Lesser eye: profound VI
Blindness (Read code)	F4915	Better eye: moderate VI, Lesser eye: blind, unspecified
Blindness (Read code)	F4916	Better eye: moderate VI, Lesser eye: total VI
Blindness (Read code)	F4917	Better eye: moderate VI, Lesser eye: near total VI
Blindness (Read code)	F4918	Better eye: moderate VI, Lesser eye: profound VI
Blindness (Read code)	F491z	One eye blind, one eye low vision NOS
Blindness (Read code)	F492	Low vision, both eyes
Blindness (Read code)	F4920	Low vision, both eyes unspecified
Blindness (Read code)	F4921	Better eye: severe VI, Lesser eye: low vision unspecified

Blindness (Read code)	F4922	Better eye: severe VI, Lesser eye: severe VI
Blindness (Read code)	F4923	Better eye: moderate VI, Lesser eye: low vision unspecified
Blindness (Read code)	F4924	Better eye: moderate VI, Lesser eye: severe VI
Blindness (Read code)	F4925	Better eye: moderate VI, Lesser eye: moderate VI
Blindness (Read code)	F492z	Low vision, both eyes NOS
Blindness (Read code)	F493	Visual loss, both eyes unqualified
Blindness (Read code)	F493-99	Blind/low vision - both eyes
Blindness (Read code)	F494	Legal blindness USA
Blindness (Read code)	F495	Profound impairment, one eye
Blindness (Read code)	F4950	Blindness, one eye, unspecified
Blindness (Read code)	F4951	Lesser eye: total visual impairment, Better eye: unspecified
Blindness (Read code)	F4952	Lesser eye: total VI, Better eye: near normal vision
Blindness (Read code)	F4953	Lesser eye: total VI, Better eye: normal vision
Blindness (Read code)	F4954	Lesser eye: near total VI, Better eye: unspecified
Blindness (Read code)	F4955	Lesser eye: near total VI, Better eye: near normal vision
Blindness (Read code)	F4956	Lesser eye: near total VI, Better eye: normal vision
Blindness (Read code)	F4957	Lesser eye: profound VI, Better eye: unspecified
Blindness (Read code)	F4958	Lesser eye: profound VI, Better eye: near normal vision
Blindness (Read code)	F4959	Lesser eye: profound VI, Better eye: normal vision
Blindness (Read code)	F495A	Acquired blindness, one eye
Blindness (Read code)	F495z	Profound impairment one eye NOS
Blindness (Read code)	F496	Low vision, one eye
Blindness (Read code)	F496-99	Blind/low vision -one eye only
Blindness (Read code)	F4960	Low vision, one eye, unspecified
Blindness (Read code)	F4961	Lesser eye: severe VI, Better eye: unspecified
Blindness (Read code)	F4962	Lesser eye: severe VI, Better eye: near normal vision
Blindness (Read code)	F4963	Lesser eye: severe VI, Better eye: normal vision
Blindness (Read code)	F4964	Lesser eye: moderate VI, Better eye: unspecified
Blindness (Read code)	F4965	Lesser eye: moderate VI, Better eye: near normal vision
Blindness (Read code)	F4966	Lesser eye: moderate VI, Better eye: normal vision
Blindness (Read code)	F496z	Low vision, one eye NOS
Blindness (Read code)	F49y	Visual loss, one eye, unqualified
Blindness (Read code)	F49y-99	Blind/low vision -one eye only
Blindness (Read code)	F49z	Visual loss NOS
Blindness (Read code)	F49z-1	Acquired blindness
Blindness (Read code)	F49z-99	Blindness/low vision NOS
Blindness (Read code)	F49z0	Charles Bonnet syndrome
Blindness (ICD10)	H54	H54 - Visual impairment including blindness (binocular or mon
Blindness (ICD10)	H540	H540 - Blindness, binocular
Blindness (ICD10)	H541	H541 - Severe visual impairment, binocular
Blindness (ICD10)	H544	H544 - Blindness, monocular
Blindness (ICD10)	H545	H545 - Severe visual impairment, monocular
Blindness (ICD10)	H549	H549 - Unspecified visual impairment (binocular)
**Group Name**	**clincial cod**	**clinical term**
Amputation (Read code)	14N4	H/O: limb amputation
Amputation (Read code)	14N4-1	Amputee - limb
Amputation (Read code)	14N41	H/O: lower limb amputation
Amputation (Read code)	14N4Z	H/O: limb amputation NOS
Amputation (Read code)	2G42	O/E - Amputated right leg
Amputation (Read code)	2G43	O/E - Amputated left leg

Amputation (Read code)	2G44	O/E - Amputated right above knee
Amputation (Read code)	2G45	O/E - Amputated left above knee
Amputation (Read code)	2G46	O/E - Amputated right below knee
Amputation (Read code)	2G47	O/E - Amputated left below knee
Amputation (Read code)	2G4A	O/E - amputated left midfoot
Amputation (Read code)	2G4B	O/E - amputated right midfoot
Amputation (Read code)	7L06	Amputation of leg
Amputation (Read code)	7L06-99	Amputation - lower limb
Amputation (Read code)	7L060	Hindquarter amputation
Amputation (Read code)	7L060-1	Ferre hindquarter amputation
Amputation (Read code)	7L060-2	Gordon - Taylor hindquarter amputation
Amputation (Read code)	7L060-3	Jaboulay hindquarter amputation
Amputation (Read code)	7L060-4	King hindquarter amputation
Amputation (Read code)	7L060-5	Sorrondo hindquarter amputation
Amputation (Read code)	7L060-6	Steelquist hindquarter amputation
Amputation (Read code)	7L060-7	Taylor hindquarter amputation
Amputation (Read code)	7L060-99	Hind quarter amputation
Amputation (Read code)	7L061	Disarticulation of hip
Amputation (Read code)	7L061-1	Boyd disarticulation of hip
Amputation (Read code)	7L061-2	Fitzmaurice - Kelly disarticulation of hip
Amputation (Read code)	7L061-99	Hip disarticulation
Amputation (Read code)	7L062	Amputation above knee
Amputation (Read code)	7L062-1	Kirk amputation of leg through thigh
Amputation (Read code)	7L062-2	Amputation of leg through thigh
Amputation (Read code)	7L062-99	Above knee amputation
Amputation (Read code)	7L063	Amputation through knee
Amputation (Read code)	7L063-1	Batch disarticulation of knee
Amputation (Read code)	7L063-2	Callander disarticulation of knee
Amputation (Read code)	7L063-3	Disarticulation of knee
Amputation (Read code)	7L063-4	Gritti-Stokes disarticulation of knee
Amputation (Read code)	7L063-5	Kirk disarticulation of knee
Amputation (Read code)	7L063-6	Mazet disarticulation of knee
Amputation (Read code)	7L063-7	McFaddin disarticulation of knee
Amputation (Read code)	7L063-8	Slocum disarticulation of knee
Amputation (Read code)	7L063-9	Spittler disarticulation of knee
Amputation (Read code)	7L063-99	Knee disarticulation
Amputation (Read code)	7L064	Amputation below knee
Amputation (Read code)	7L064-1	Boyd amputation of leg below knee
Amputation (Read code)	7L064-2	Burgess amputation of leg below knee
Amputation (Read code)	7L064-3	Guyon amputation of leg below knee
Amputation (Read code)	7L064-98	Below knee amputation
Amputation (Read code)	7L064-99	Supramalleolar ankle amputat.
Amputation (Read code)	7L06y	Other specified amputation of leg
Amputation (Read code)	7L06z	Amputation of leg NOS
Amputation (Read code)	7L06z-99	Amputation lower limb NOS
Amputation (Read code)	7L07	Amputation of foot
Amputation (Read code)	7L070	Amputation through ankle
Amputation (Read code)	7L070-1	Pirogoff amputation of foot through ankle
Amputation (Read code)	7L070-2	Syme amputation of foot through ankle
Amputation (Read code)	7L070-99	Disarticulation of foot

Amputation (Read code)	7L071	Disarticulation of tarsal bones
Amputation (Read code)	7L071-1	Boyd amputation of hindfoot
Amputation (Read code)	7L071-99	Amputation foot: mid-tarsal
Amputation (Read code)	7L072	Disarticulation tarsometatarsal joint
Amputation (Read code)	7L072-1	Lisfranc tarsometatarsal amputation
Amputation (Read code)	7L072-2	Tarsometatarsal amputation
Amputation (Read code)	7L072-3	Disarticulation of metatarsal bones
Amputation (Read code)	7L072-99	Amputation foot:tarsal-metatar
Amputation (Read code)	7L073	Amputation through metatarsal bones
Amputation (Read code)	7L073-1	Chopart midtarsal amputation
Amputation (Read code)	7L073-2	Ray transmetatarsal amputation
Amputation (Read code)	7L07y	Other specified amputation of foot
Amputation (Read code)	7L07z	Amputation of foot NOS
Amputation (Read code)	7L07z-1	Hey amputation of foot
Amputation (Read code)	7L08	Amputation of toe
Amputation (Read code)	7L080	Amputation hallux
Amputation (Read code)	7L080-1	Amputation great toe
Amputation (Read code)	7L081	Amputation of phalanx of toe
Amputation (Read code)	7L082	Proximal hemiphalangectomy of toe
Amputation (Read code)	7L083	Amputation lesser toe
Amputation (Read code)	7L084	Terminalisation of hallux
Amputation (Read code)	7L085	Terminalisation of lesser toe
Amputation (Read code)	7L08y	Other specified amputation of toe
Amputation (Read code)	7L08z	Amputation of toe NOS
Amputation (Read code)	7L08z-1	Disarticulation of toe NOS
Amputation (OPCS-4 code)	X093	X093 - Amputation of leg above knee
Amputation (OPCS-4 code)	X094	X094 - Amputation of leg through knee
Amputation (OPCS-4 code)	X095	X095 - Amputation of leg below knee
Amputation (OPCS-4 code)	X098	X098 - Other specified amputation of leg
Amputation (OPCS-4 code)	X099	X099 - Unspecified amputation of leg
Amputation (OPCS-4 code)	X10	X10 - Amputation of foot
Amputation (OPCS-4 code)	X101	X101 - Amputation of foot through ankle
Amputation (OPCS-4 code)	X104	X104 - Amputation through metatarsal bones
Amputation (OPCS-4 code)	X108	X108 - Other specified amputation of foot
Amputation (OPCS-4 code)	X109	X109 - Unspecified amputation of foot
Amputation (OPCS-4 code)	X11	X11 - Amputation of toe
Amputation (OPCS-4 code)	X111	X111 - Amputation of great toe
Amputation (OPCS-4 code)	X112	X112 - Amputation of phalanx of toe
Amputation (OPCS-4 code)	X118	X118 - Other specified amputation of toe
Amputation (OPCS-4 code)	X119	X119 - Unspecified amputation of toe
